# Corticospinal Excitability of the Lower Limb Muscles During the Anticipatory Postural Adjustments: A TMS Study During Dart Throwing

**DOI:** 10.3389/fnhum.2021.703377

**Published:** 2021-10-28

**Authors:** Amiri Matsumoto, Nan Liang, Hajime Ueda, Keisuke Irie

**Affiliations:** Cognitive Motor Neuroscience, Human Health Sciences, Graduate School of Medicine, Kyoto University, Kyoto, Japan

**Keywords:** postural control, center of pressure (COP), transcranial magnetic stimulation, motor evoked potential (MEP), central command, three-dimensional motion analysis, motor imagery ability

## Abstract

**Objective:** To investigate whether the changes in the corticospinal excitability contribute to the anticipatory postural adjustments (APAs) in the lower limb muscles when performing the ballistic upper limb movement of the dart throwing.

**Methods:** We examined the primary motor cortex (M1) excitability of the lower limb muscles [tibialis anterior (TA) and soleus (SOL) muscles] during the APA phase by using transcranial magnetic stimulation (TMS) in the healthy volunteers. The surface electromyography (EMG) of anterior deltoid, triceps brachii, biceps brachii, TA, and SOL muscles was recorded and the motor evoked potential (MEP) to TMS was recorded in the TA muscle along with the SOL muscle. TMS at the hotspot of the TA muscle was applied at the timings immediately prior to the TA onset. The kinematic parameters including the three-dimensional motion analysis and center of pressure (COP) during the dart throwing were also assessed.

**Results:** The changes in COP and EMG of the TA muscle occurred preceding the dart throwing, which involved a slight elbow flexion followed by an extension. The correlation analysis revealed that the onset of the TA muscle was related to the COP change and the elbow joint flexion. The MEP amplitude in the TA muscle, but not that in the SOL muscle, significantly increased immediately prior to the EMG burst (100, 50, and 0 ms prior to the TA onset).

**Conclusion:** Our findings demonstrate that the corticospinal excitability of the TA muscle increases prior to the ballistic upper limb movement of the dart throwing, suggesting that the corticospinal pathway contributes to the APA in the lower limb in a muscle-specific manner.

## Introduction

Perturbations from voluntary movements such as the reaching or unloading of the upper limb cause the shift of the center of gravity (COG) and impair the postural equilibrium in humans (Aruin and Latash, 1995a). The activities of the postural muscles in the trunk and lower limb occur prior to a voluntary upper limb movement (Kasai and Taga, 1992; Kawanishi et al., 1999; Chiou et al., 2018), which is known as the anticipatory postural adjustments (APAs). The APA is thought to contribute to the postural control, which minimizes the postural displacement from an expected perturbation in advance and plays an important role in maintaining balance and preventing falls (Horak, 2006; Kanekar and Aruin, 2014a). The abnormal APA has been shown in elderly people (Kanekar and Aruin, 2014a,b) and in the patients with stroke (Palmer et al., 1996; Garland et al., 1997; Slijper et al., 2002; Bourke et al., 2015), cerebral palsy (Bigongiari et al., 2011; Girolami et al., 2011), Parkinson's disease (Viallet et al., 1987; Latash et al., 1995), multiple sclerosis (Krishnan et al., 2012; Aruin et al., 2015), and chronic low back pain (Hodges and Richardson, 1996; Massé-Alarie et al., 2012).

It has been reported that the preceding activity of the postural muscles in association with the APA was affected by the velocity of the intended movements (Lee et al., 1987). This is explained as a rapid shoulder movement causes a perturbation and the preceding activities of the postural muscles allow to minimize the postural instability in advance, while a slow shoulder movement causes minimum perturbation and needs no postural control in advance. The preceding postural control is observed approximately 100 ms before the initiation of the intended movement (Aruin and Latash, 1995b). Because the time window of the APA is too fast as a result of the afferent inputs from the upper limb movement, it is thought to be preprogrammed by the central nervous system (CNS) (Friedli et al., 1984; Massion, 1992).

Although the several cortical and subcortical mechanisms, involving the primary motor cortex (M1), supplementary motor area (SMA), basal ganglia, thalamus, brainstem, vestibule, and spinal cord, are thought to contribute to the APA (Viallet et al., 1992; Jacobs et al., 2009; Ng et al., 2011, 2013), the cortical contribution rather than the subcortical contribution might have a greater role (Massion, 1992; Chiou et al., 2018). To investigate cortical or corticospinal excitability, transcranial magnetic stimulation (TMS) has been widely utilized to date (Barker et al., 1985). The advantage of TMS is not only capable of stimulating the cerebral cortex non-invasively, but also of targeting the area in the M1 responsible for the control of a specific muscle. By using TMS, it has been shown that the corticospinal excitability increased in the lower limb and trunk muscles in the preparation of the rapid shoulder and elbow movements (Petersen et al., 2009; Chiou et al., 2016, 2018; Massé-Alarie et al., 2018).

The previous studies suggest that M1 may contribute to the APA, while the central mechanisms of the APA are not fully understood. Particularly, it remains unclear whether the corticospinal tract for the tibialis anterior (TA) muscle in the lower limb is involved in the APA when performing a ballistic movement of the upper limb. Because it has been shown that the motoneurons of the TA muscle receive a greater excitatory influence from the M1 compared to the antigravity muscle of the triceps surae muscle within the lower limb (Brouwer and Ashby, 1992; Bawa et al., 2002), it is expected that the excitability of the corticospinal projections to the TA muscle increases in the APA phase as well as that observed in the triceps surae muscle. We also believed that it would be of great interest to investigate a ballistic multijoint movement with more intended and goal-directed action, e.g., dart throwing. It is considered that the APA operates for the throwing movement involving a slight flexion of the elbow joint followed by an extension, which is poorly understood. Furthermore, whether the changes in the corticospinal excitability, if any, correlate to the outcome of the cognitive characteristics, namely, the changes in the kinematic parameters in association with the voluntary movement or the individual motor imagery ability, are considered in the scope of this study. It has been shown that the motor imagery accompanies increments of the cortical excitability including the M1 (Yahagi et al., 1996; Kasai et al., 1997). If the corticospinal tract contributes to the APA as we hypothesized, the corticospinal excitability may be modulated depending on the optimal attentional strategy of an individual, which is related to the modality dominance of the motor imagery (Sakurada et al., 2016).

We, therefore, hypothesized that the corticospinal pathway contributes to the APA in the lower limb preceding the ballistic upper limb movement. To test this hypothesis, we used TMS to examine the changes in the excitability of the corticospinal projections to the TA muscle in the time window of the APA phase during the dart throwing. Also, we assessed the kinematic parameters by means of the three-dimensional motion analysis, center of pressure (COP), and the individual visual and kinesthetic motor imagery abilities (Malouin et al., 2007).

## Materials and Methods

### Participants

A total of 17 right-handed [the Flinders Handedness survey (FLANDERS) questionnaire, 8.8 ± 2.7 points] (Nicholls et al., 2013; Okubo et al., 2014) healthy volunteers, who did not suffer from any known neurological or orthopedic disorders and did not have any prescribed medication or CNS active drugs, participated in this study. Fifteen (six men and nine women; mean age 24 ± 4 years) of the participants were recruited in protocol 1, of which nine participants were additionally assessed by the three-dimensional motion analysis. Thirteen (five men and eight women; mean age 24 ± 4 years) of 15 participants who participated in protocol 1 were also in protocol 2. Seven (two men and five women; mean age 25 ± 4 years) of the participants were recruited in protocol 3, of which five participants participated in both protocols 1 and 2. All the participants, who were non-professional dart players, gave their informed written consent before the experiments. The experimental procedures and protocols were performed in accordance with the Declaration of Helsinki and approved by the Ethics Committee of Kyoto University Graduate School and Faculty of Medicine.

### Experimental Procedures

The participants were asked to stand upright on the throwing line (on a force plate) with their feet closed and face the dart board straight. A plastic competition dart board (diameter: 39.4 cm) was set in front of the participant, 220 cm from the throwing line and 173 cm off the ground. In the preparative position, the participants were asked to hold a plastic tip dart (18 g) with the right dominant hand when keeping the right shoulder and elbow joint flexed and then to throw the dart after a visual go signal [light-emitting diode (LED) light], which was set beneath the dart board. The Participants were instructed to “keep standing in an upright position without moving as much as possible when holding the dart, then aim the center of the board (bulls-eye) and throw the dart as forcefully as possible by means of right elbow extension movement after the visual cue.” The non-dominant arm and hand were relaxed throughout the experiment. About 5 to 10 familiarization trials were performed prior to the data recordings to familiarize the participants with the task.

### Measurements of the Motor Performance

The kinematic assessments by the three-dimensional motion analysis were performed by using the KinemaTracer system (Motion Recorder, KISSEI COMTEC Corporation Ltd., Japan) of which the four cameras were set in an equidistant manner on the right side of the participant (sampling rate 50 Hz). Eight reflective markers were placed on the right acromion, lateral epicondyle approximating elbow joint axis, ulnar styloid, fifth metacarpal head, greater trochanter, lateral epicondyle of the knee, lateral malleolus, and fifth metatarsal head according to the Plug-in-Gait marker placement. The real-time angle changes in the shoulder, elbow, and wrist joints in the right upper limb and those in the hip, knee, and ankle joints in the right lower limb were recorded. COP was recorded throughout the experiment by a force plate (90 cm × 60 cm, TF-6090, Tec Gihan Corporation Ltd., Japan) set under the feet of the participant.

### Electromyography Recordings

Surface EMG was recorded from the right anterior deltoid (AD), long head of triceps brachii (TB), TA, and soleus (SOL) muscles by using a pair of the silver-bar electrodes (10 mm in length, 1 mm in diameter, and 10 mm in distance, Bagnoli-4 EMG System, Delsys, Boston, Massachusetts, USA) attached on the muscle belly closely to the predicted neuromuscular junction of each muscle. The reference electrode was attached to the right olecranon. The AD and TB muscles are thought to contribute to dart throwing, while the TA and SOL muscles are thought to contribute to postural control. To confirm the contribution of the biceps brachii (BB) muscle to the APA, we recorded EMG activity of the BB muscle instead of the AD muscle in protocol 3. The EMG signals were amplified (1,000X) and passed through a bandpass filter between 20 and 2,000 Hz.

### Transcranial Magnetic Stimulation

**Figure 1:**
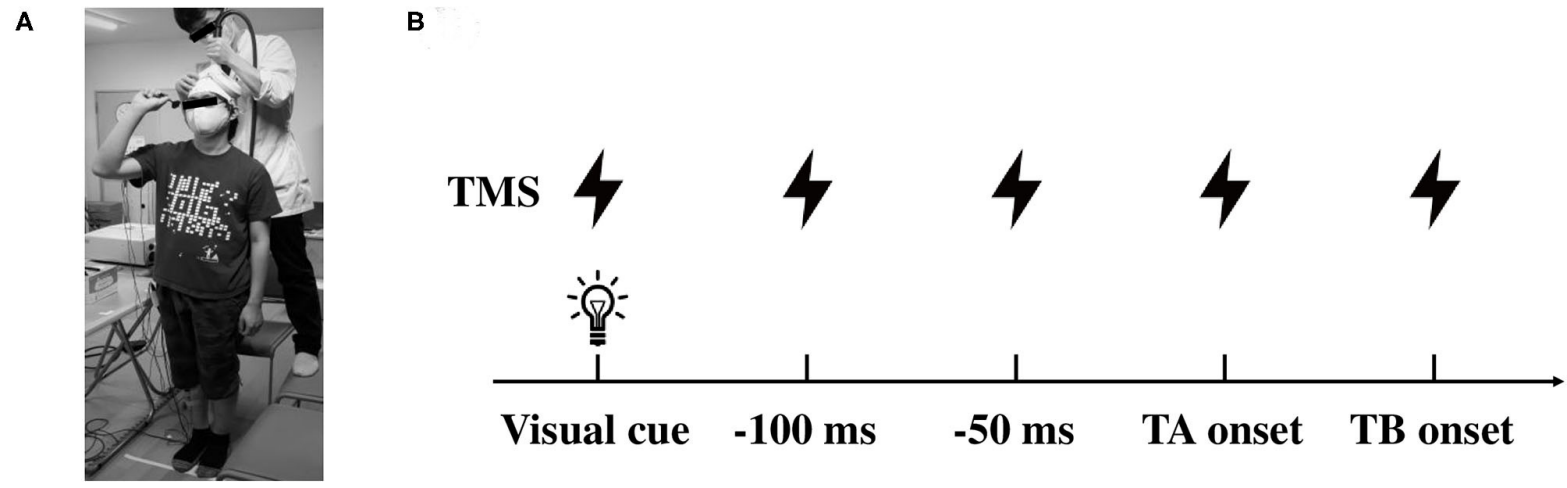
Illustrations of the experimental setup. **(A)** Data collection of the motor evoked potential (MEP) in the lower limb muscles during dart throwing. **(B)** The timing of TMS application in protocol 2. TMS was applied at the visual cue, 100, 50, 0 ms prior to the TA onset and the TB onset. TMS, transcranial magnetic stimulation; TA, tibialis anterior; TB, triceps brachii.

A double cone coil (13 cm external diameter of wings) connected to a magnetic stimulator (Magstim 200 square, The Magstim Company Ltd., Whitland, UK) was placed around the vertex (Figure 1A). The coil current was applied in an anterior-posterior direction with the coil loops lateral to the midline and, therefore, a monophasic current with a posterior-anterior direction was applied in the M1. The center of the junction of the coil was systematically adjusted to find the optimum location for the activation of the right TA muscle, which was 1 cm lateral and 1 cm anterior to the vertex. We determined the optimal position (motor hotspot) where stimulation of the slight suprathreshold intensity consistently produced the largest motor evoked potential (MEP) in the right TA muscle by moving the coil in 0.5 cm and the motor hotspot was marked with a pen on the swimming cap covered scalp. The resting motor threshold (rMT) was defined as the lowest stimulus intensity of TMS evoking MEP of above 50 μV in amplitude in more than half of the trials. The stimulus intensity was set at 1.1–1.2 times rMT (55 ± 8, 39–68% of the maximum stimulator output) for inducing a definitely identifiable MEP (approximately 0.2–0.4 mV in the resting state) in the experiments. TMS pulse was delivered by a three-channel electronic stimulator (SEN-7203, Nihon Kohden, Tokyo, Japan) by which the visual cue (LED light) was triggered synchronously.

### Experimental Protocols

Three experimental protocols were carried out in this study. In protocol 1, participants performed the dart throwing with a visual cue triggered reaction time paradigm without TMS. The timing of visual cues was randomized, and the interval between the cues was approximately 15–30 s. One session included five trials and the time interval between the sessions was approximately 3–5 min. Participants performed 10 trials (two sessions) along with the recordings of the motor performance of the distances from the thrown darts to the bull's-eye, the three-dimensional motion analysis focused on the right upper and lower limb movements, and the COP. The EMG onset timing (interval from the visual cue to the EMG onset) of all four muscles was calculated.

In protocol 2, TMS was given at several time points, which were predetermined by the analysis in protocol 1. Because the EMG of the TA muscle was clearly observed prior to the EMG onset of the TB muscle (agonist muscle), TMS was applied at the timings of the visual cue, 0, 50, and 100 ms prior to the EMG onset of the TA muscle (TA onset, −50 ms, and −100 ms, respectively) and also the EMG onset of the TB muscle (TB onset, Figure 1B). Because of the difference in the timing of the movement initiation following the visual cue among the participants, the trigger timing of TMS was determined in each participant (total of 13 participants). It has been shown that the corticospinal excitability of the muscles involved in a motor task increased from about 100 ms prior to the EMG onset (Chen and Hallett, 1999). With respect to the APA, a previous study also reported that the corticospinal excitability increased 75 ms prior to the EMG onset of the postural muscle in the lower limb (Petersen et al., 2009). In this study, therefore, we aimed to explore the changes in the corticospinal excitability in the APA time window during the dart throwing and chose the timings immediately before the TA muscle bursts. The timing of the TB onset was chosen to explore the extent to the increment of the MEP amplitude at the moment of the agonist EMG onset, while the timing of the visual cue was chosen to confirm whether the changes in MEP were task-dependent. At least five trials (5–10 trials) were conducted at each time point. The EMG activity of the SOL muscle as an antigravity muscle and that of the AD muscle as an adjunctive muscle were frequently presented before the dart throwing because the participants held a dart with their right shoulder and elbow flexed in a standing position as mentioned above. Focusing on the TA muscle in this study, we carefully confirmed, throughout the experiments and the offline analysis, that no EMG activity in the TA muscle at the time points TMS applied, except the time point of the TB onset. In the control condition, TMS was applied while the participants were standing upright in the resting state without holding a dart.

In protocol 3, we additionally recorded the EMG activity of the BB muscle instead of the AD muscle because the movement of the elbow flexion might play a role at the early stage of the APA according to the kinematic data. The experimental procedures were the same as in protocol 1.

Apart from the main protocols, the motor imagery abilities were assessed by using the Kinesthetic and Visual Imagery Questionnaire (KVIQ), which was developed to determine the individual visual imagery (VI) and kinesthetic imagery (KI) abilities, respectively (Malouin et al., 2007). Because it has been shown in the previous study that the motor performance outcome can be affected by the optimal attentional strategy of an individual, which is related to the modality dominance of the motor imagery (Sakurada et al., 2016), we aimed to explore the difference in the VI and KI in our participant group and, if any, the influence on the resulting motor performance or the corticospinal excitability.

### Data Acquisition and Analysis

The linear distances from the bull's-eye to the thrown darts were measured as the results of the motor performance.

During the dart throwing task, the changes in the angle and angular velocity of the shoulder, elbow, and wrist joints in the right upper limb and those in the hip, knee, and ankle joints in the right lower limb were calculated (3D Calculator, KISSEI COMTEC Corporation Ltd., Japan). The force plate signal (force and its vectors in the axial directions of the x, y, and z-axes) was sampled at 50 Hz (Vital Recorder 2, KISSEI COMTEC Corporation Ltd., Japan) and the data were stored in a computer for the offline analysis (Kine Analyzer, KISSEI COMTEC Corporation Ltd., Japan). The total length of COP and rectangle area for 3 s from the visual cue were calculated.

The EMG activities were recorded and analyzed by using the data acquisition software (LabChart, ADInstruments, Sydney, Australia) for the PowerLab analog-to-digital convertor (PowerLab 8/30, AD Instruments, Sydney, Australia) at a 4-kHz sampling rate. EMG signals were rectified and analyzed with a moving average of 50 ms without TMS. The interval from the visual cue to the EMG onset of each muscle and the kinematic parameters was calculated, respectively. All the time course data were also realigned to the TB muscle onset (defined as 0 ms). The EMG activity before the visual cue (with a 100 ms window) was calculated and the value of mean ± 2 SD in each participant was used as a cutoff value to determine the onset and end of the EMG activities followed by the visual inspection of the experimenters. EMG activity with the maximum voluntary contraction (MVC) of each muscle was recorded at the beginning of the experiments. Participants were asked to maximally perform the shoulder flexion, elbow flexion and extension, and dorsal and plantar flexion for 2–3 s, and the MVC per second was calculated for each muscle. MVC for plantar flexion was measured in a standing position, while that for others in a sitting position. The integrated EMG (iEMG) activities were calculated and the averaged values per second during the motor tasks were presented as a percentage of MVC (%MVC).

The MEP amplitude was measured as the peak-to-peak values and normalized as a percentage of MEP at the control condition (%control). The background EMG (bEMG) activities prior to the TMS trigger (with a 100 ms window) were calculated in all the trials. The trials including significant bEMG activity in the TA muscle were excluded, except at the time point of the TB muscle in which almost all the trials contained bEMG activity (these data were all included in the analysis). After omitting the trials involving significant bEMG activity in the TA muscle (time points of visual cue, −100 ms, −50 ms, and the TA onset), the number for the control condition involved in the analysis was 8.7 ± 1.2 trials, while that for the time points during the motor task was 4.9 ± 2.3 trials.

The motor performance and kinematic data with TMS (protocol 2) were not utilized in the analysis because they would be markedly influenced by the preceding TMS of which the stimulation would spread in the M1 and induce muscle activation not only in the TA and the SOL muscles but also in the other muscles (e.g., upper limb and trunk muscles).

### Statistical Analysis

Data were analyzed by using the JMP Pro 15 software (SAS Institute Incorporation, Cary, North Carolina, USA). In protocols 1 and 3, the one-way ANOVA with repeated measures (factor: muscle) was used to determine the difference in the EMG onset timing followed by the Dunnett's *post-hoc* test. The timing between EMG onset and the onset of the COP displacement or elbow joint movement was analyzed with a paired *t*-test. The KVIQ score was analyzed with the Wilcoxon signed-rank test. The correlation analysis between the EMG onsets or kinematic data was performed by using Pearson's correlation coefficient analysis. In protocol 2, the MEPs in the TA and SOL muscles were normalized as a ratio of the control size (resting standing), and then grand mean ratios with SD from the pooled data were calculated. These data were analyzed by using the one-way ANOVA with the repeated measures (factor: time point) followed by a paired *t*-test with the Holm's Sequential Bonferroni Correction (Holm, 1979). The MEP amplitude at the TB onset and at the control was compared with a paired *t*-test. The correlation analysis of the changes in the MEP of the TA or SOL muscle with the KVIQ scores was performed by using Spearman's rank correlation. The level of the statistical significance was defined as *p* < 0.05. Results are presented as mean ± SD. The effect size for the ANOVA was calculated by using eta squared (η^2^) (Cohen, 1988).

## Results

### Electromyography Activity

**Figure 2:**
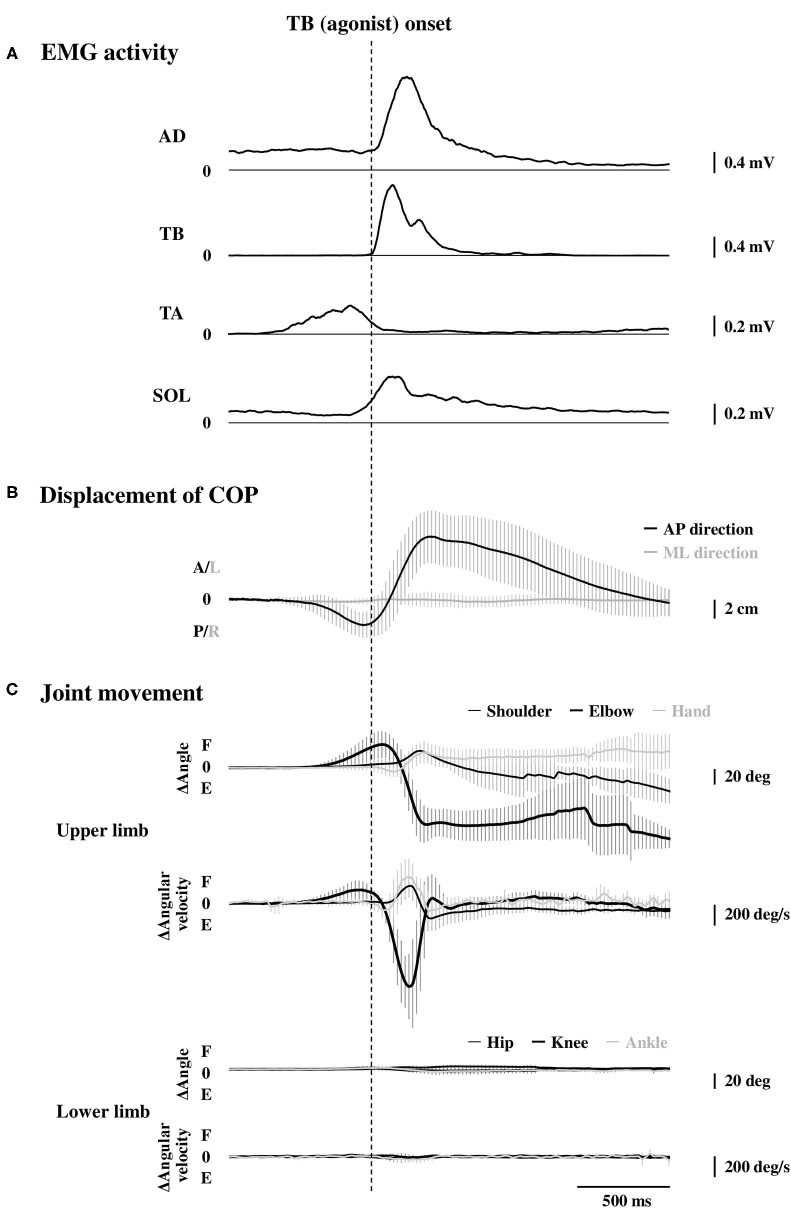
Rectified and averaged EMG activities of the AD, TB, TA, and SOL muscles (**A**, *n* = 15), displacement of the COP (**B**, *n* = 15), and the joint movements in the upper and lower limbs (**C**, *n* = 9) in the time course. All the data were aligned to the EMG onset of the TB muscle. Error bar indicates the SD. EMG, electromyography; AD, anterior deltoid; TB, triceps brachii; TA, tibialis anterior; SOL, soleus; COP, the center of pressure.

Averaged data of the EMG activity, which is aligned to the TB onset in the time course, are shown in Figure 2A (*n* = 15). The iEMG of the AD, TB, TA, and SOL muscles during the dart throwing was 55.8 ± 32.0%, 52.7 ± 22.8%, 12.7 ± 7.0%, and 29.5 ± 26.6% MVC, respectively. The EMG onset timing was obviously different among the muscles [*F*_(3.42)_ = 76.74, *p* < 0.0001, η^2^ = 0.80]; the EMG activity of the TA muscle significantly preceded the agonist TB muscle onset (*p* < 0.0001, Table 1), while the onset of the AD muscle was significantly delayed (*p* < 0.001). The EMG of the SOL muscle occurred slightly earlier (but not significant) compared to the TB muscle (*p* = 0.13). In addition, the onset of the BB muscle was significantly different compared to the other muscles [*F*_(3.18)_ = 37.89, *p* < 0.0001, η^2^ = 0.83; *post-hoc* test, *p* < 0.05, respectively, Table 1 and **Figure 4A**, *n* = 7] and the time intervals between the BB muscle and the TB, TA, and SOL muscles were 284.1 ± 98.6 ms, −42.4 ± 13.4 ms, and 283.7 ± 78.5 ms, respectively (realigned to the onset of the BB muscle).

**Table 1:** EMG and the onset of the kinematic parameters are aligned to the TB onset.

	**Time [ms]**
**EMG**	
BB	−284.1 ± 98.6
AD	50.1 ± 44.3
TA	−323.3 ± 127.9
SOL	−26.2 ± 62.5
**Displacement of COP**	
COP-posterior	−319.5 ± 126.5
COP-anterior	−96.8 ± 90.6
**Elbow joint movements**	
Elbow flexion	−284.4 ± 111.1
Elbow extension	17.3 ± 33.4

*Values are mean ± SD*.

*EMG, electromyography; TB, triceps brachii; BB, biceps brachii; AD, anterior deltoid; TA, tibialis anterior; SOL, soleus; COP, the center of pressure*.

### Motor Performance

The errors calculated by the distances from the bull's-eye to the thrown darts were 9.8 ± 3.0 cm without TMS. The average changes in the displacement of the COP in the time course are shown in Figure 2B (*n* = 15). Although the COP showed a minimum change in the left-right direction, it moved slightly in the posterior direction initially (Table 1) and then switched to the anterior direction, significantly preceding the TB onset (*p* < 0.0001, respectively). The TA muscle was activated simultaneously with the posterior shift of the COP followed by the anterior shift of the COP. The total length of the COP was 32.0 ± 9.0 cm and the rectangle area of the COP was 39.9 ± 19.6 cm^2^ during the dart throwing.

The maximum changes in the angle and angular velocity of all the joint movements are summarized in Table 2 and the average changes in these parameters in the time course are shown in Figure 2C (*n* = 9). In this study, the flexion movements in the upper limb joints were approximately 30°, while the extension movement in the elbow joint achieved the full range of 90° along with the highest angular velocity. In the time course, the elbow flexion initiated prior to the TB muscle onset (*p* < 0.0001, Table 1) followed by an extension movement along with the TB muscle onset (*p* = 0.16). In the lower limb, on the other hand, the changes in the hip, knee, and ankle joints, which were always detected after the TB onset, were minimal, if any (<5° in average). We confirmed by these data that no obvious movement of the lower limb or trunk throughout the motor task, especially prior to the TB onset.

**Table 2:** Kinematic information during the dart throwing.

**Joint**	**Movement**	**ΔAngle [deg]**	**ΔAngular velocity [deg/s]**
Shoulder	Flexion	26.6 ± 9.7	317.0 ± 98.7
	Extension	1.4 ± 2.0	211.9 ± 114.3
Elbow	Flexion	33.8 ± 17.0	160.7 ± 80.2
	Extension	90.4 ± 18.6	1204.6 ± 220.7
Hand	Palmar flexion	28.9 ± 11.8	504.1 ± 142.2
	Dorsal flexion	12.2 ± 7.0	390.6 ± 104.3
Hip	Flexion	4.3 ± 2.5	38.4 ± 14.2
	Extension	0.8 ± 0.7	23.7 ± 9.3
Knee	Flexion	2.9 ± 2.9	34.8 ± 13.8
	Extension	4.0 ± 3.2	52.2 ± 35.6
Ankle	Dorsal flexion	2.6 ± 1.5	50.5 ± 22.8
	Plantar flexion	4.9 ± 3.9	86.5 ± 53.2

*Values are mean ± SD*.

**Figure 3:**
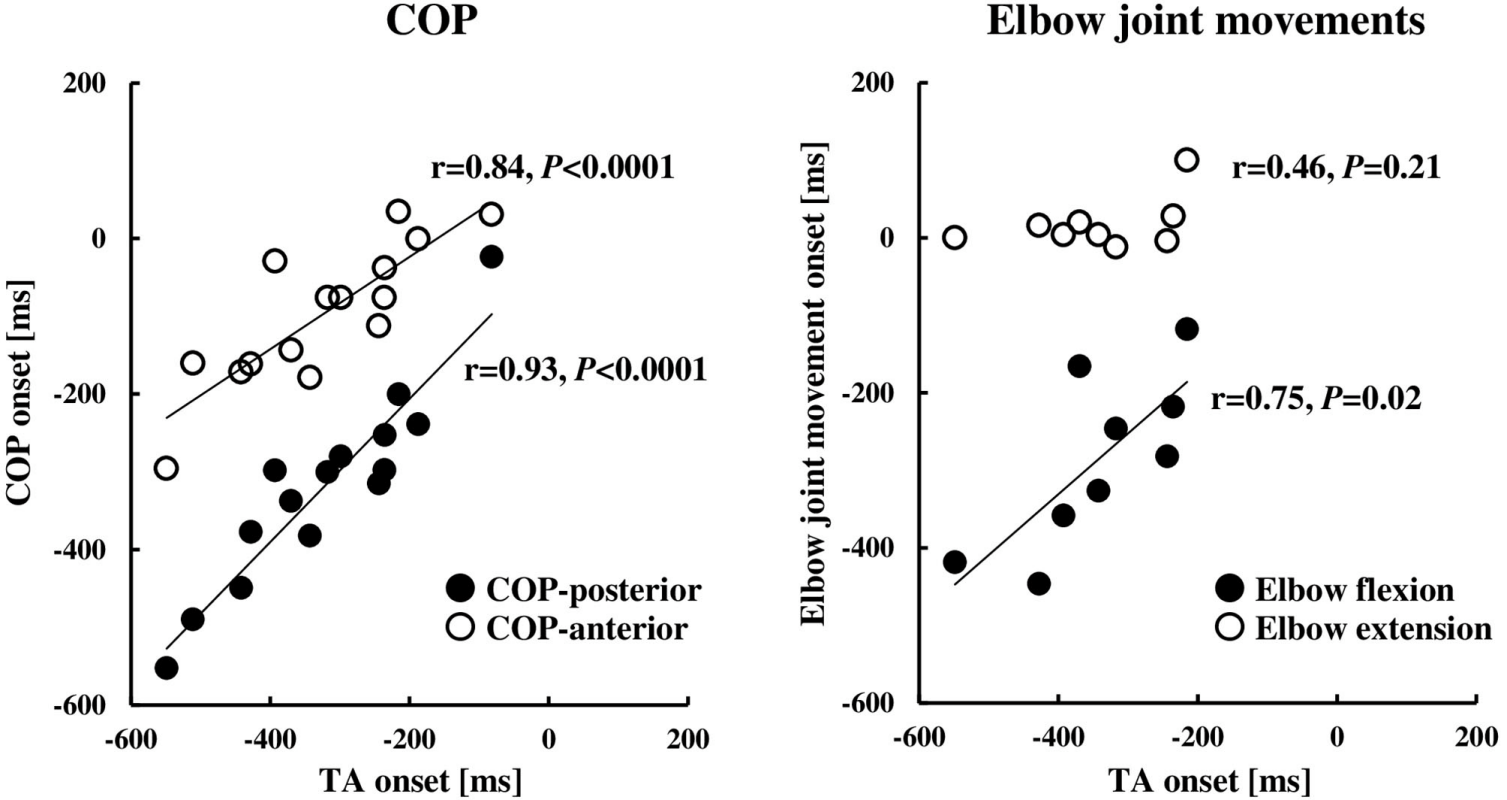
Relationships between the onset of the TA muscle and COP (*n* = 15) or elbow joint movement (*n* = 9). The onset timings were calculated by the onset of the TB muscle (0 ms, the TB onset). COP, the center of pressure; TA, tibialis anterior; TB, triceps brachii.

**Figure 4:**
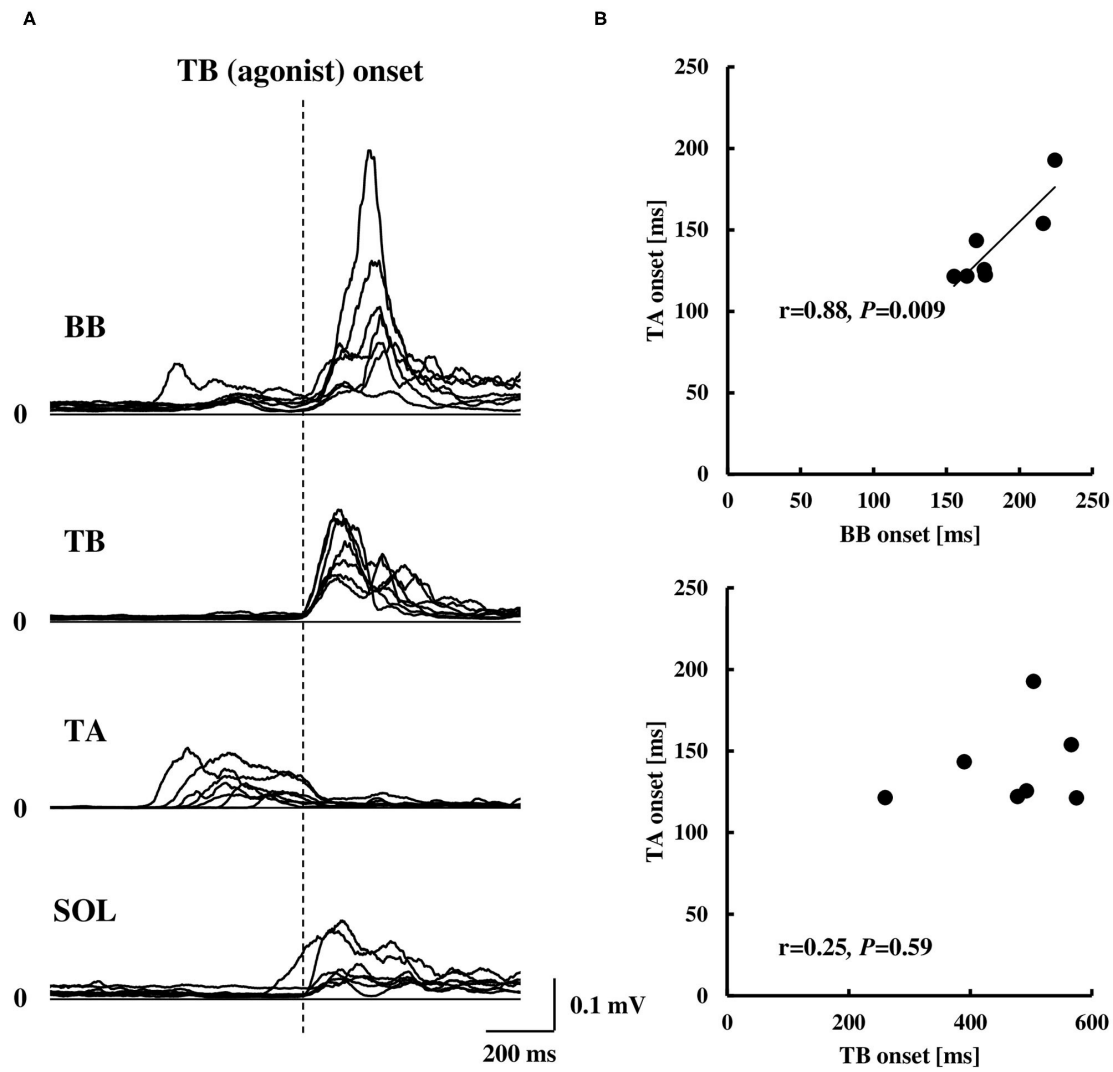
Rectified EMG activities of the BB, TB, TA, and SOL muscles aligned to the EMG onset of the TB muscle (**A**, *n* = 7) and correlation of the EMG onset of the TA muscle compared to the BB or TB muscle (**B**, *n* = 7). Note that the EMG onset in **(B)** was calculated by the duration from visual cue to the EMG onset of each muscle. BB, biceps brachii; TB, triceps brachii; TA, tibialis anterior; SOL, soleus.

The COP or elbow movement onset timing against the TA onset was assessed, respectively (Figure 3). There was a significant positive correlation between the onset of the TA and the onset of the COP posterior or anterior shift. On the other hand, the TA onset was positively correlated to the onset of the elbow flexion, but not in the case of the elbow extension. The results were in line with those in protocol 3 that the onset of the BB muscle, but not the TB muscle, showed a significant positive correlation to the onset of the TA muscle (Figure 4B).

### Motor Evoked Potential to TMS

**Figure 5:**
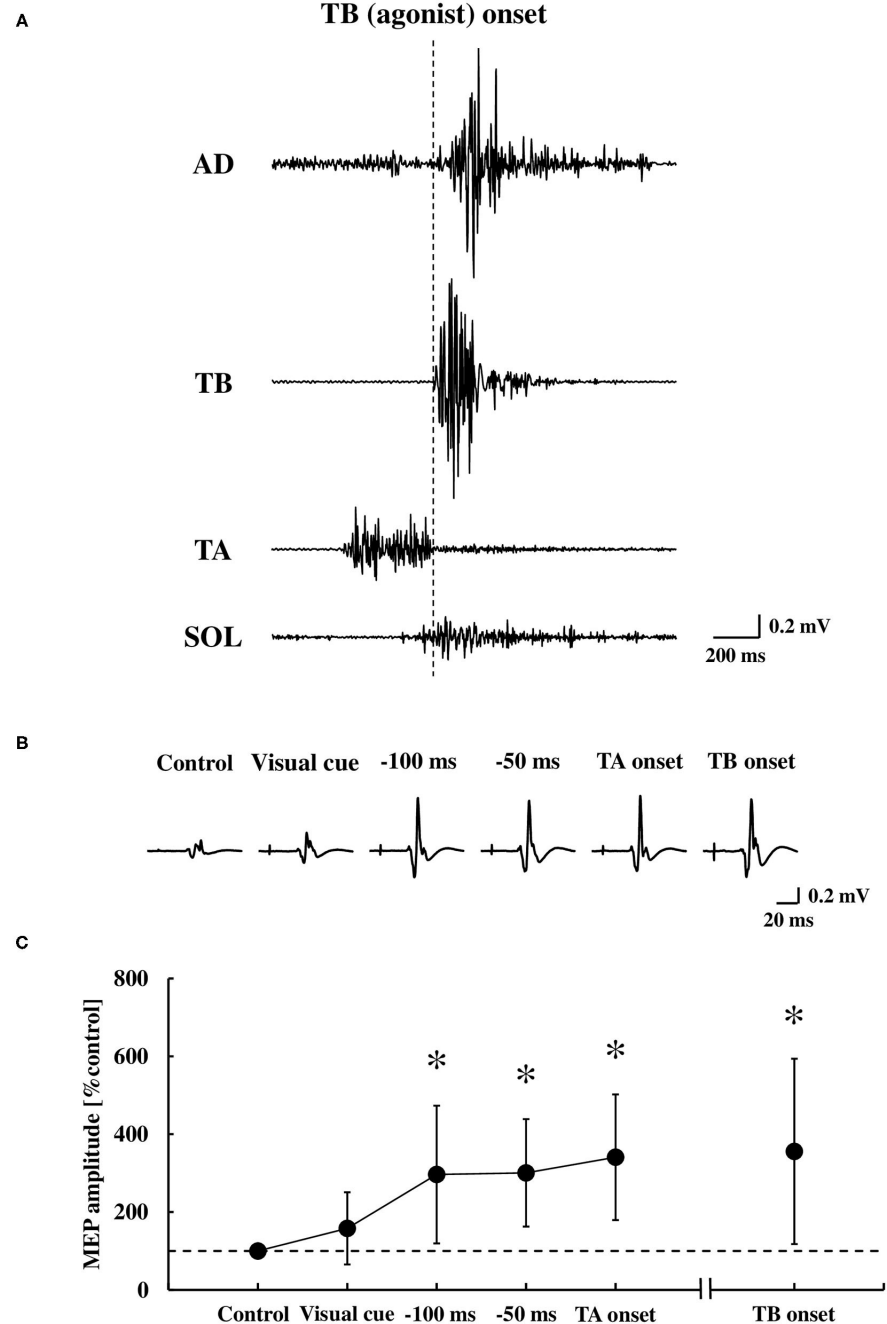
Original tracings demonstrating the AD, TB, TA, and SOL muscle in the EMG activities during the dart throwing **(A)**, representative recordings of the MEP in the TA muscle (averaged five trials, respectively) **(B)**, and the average changes at the time points tested **(C)** (*n* = 13). Error bar indicates the SD. *p < 0.01 significant difference from the control or visual cue. AD, anterior deltoid; TB, triceps brachii; TA, tibialis anterior; SOL, soleus; MEP, motor evoked potential.

Representative EMG activities and MEP recordings in the TA muscle are shown in Figures 5A,B, respectively. There was a significant difference in the MEP of the TA muscle between the time points [*F*_(4.48)_ = 8.28, *p* < 0.0001, η^2^ = 0.36, Figure 5C]. A *post-hoc* analysis revealed that the MEP in the TA muscle significantly increased at −100 ms, −50 ms, and the TA onset, but not at the visual cue, compared to the control (0.28 ± 0.23 mV, *p* < 0.01, respectively). The MEP prior to the TA onset (−100 ms, −50 ms, and the TA onset) was also significantly larger compared to the visual cue (*p* < 0.01, respectively). The MEP at the TB onset compared to the control also showed a significant increase (*p* < 0.01), in which the MEPs with bEMG activities were included.

Although the MEP was recorded by TMS over the hotspot of the TA muscle, the MEP in the SOL muscle was also obtained simultaneously in 12 out of 13 participants. At these time points relative to the TA muscle onset, we found no changes in the MEP of the SOL muscle at the visual cue (124.8 ± 59.5% control), −100 ms (108.1 ± 45.1% control), −50 ms (98.6 ± 42.9% control), and the TA onset (87.1 ± 45.5% control) compared to the control size (0.23 ± 0.18 mV) [the one-way ANOVA, *F*_(4.44)_ = 1.24, *p* = 0.31, η^2^ = 0.08], while the MEP significantly increased at the TB onset (284.3 ± 205.8% control, *p* < 0.05).

### Kinesthetic and Visual Imagery Questionnaire and Motor Evoked Potential

**Figure 6:**
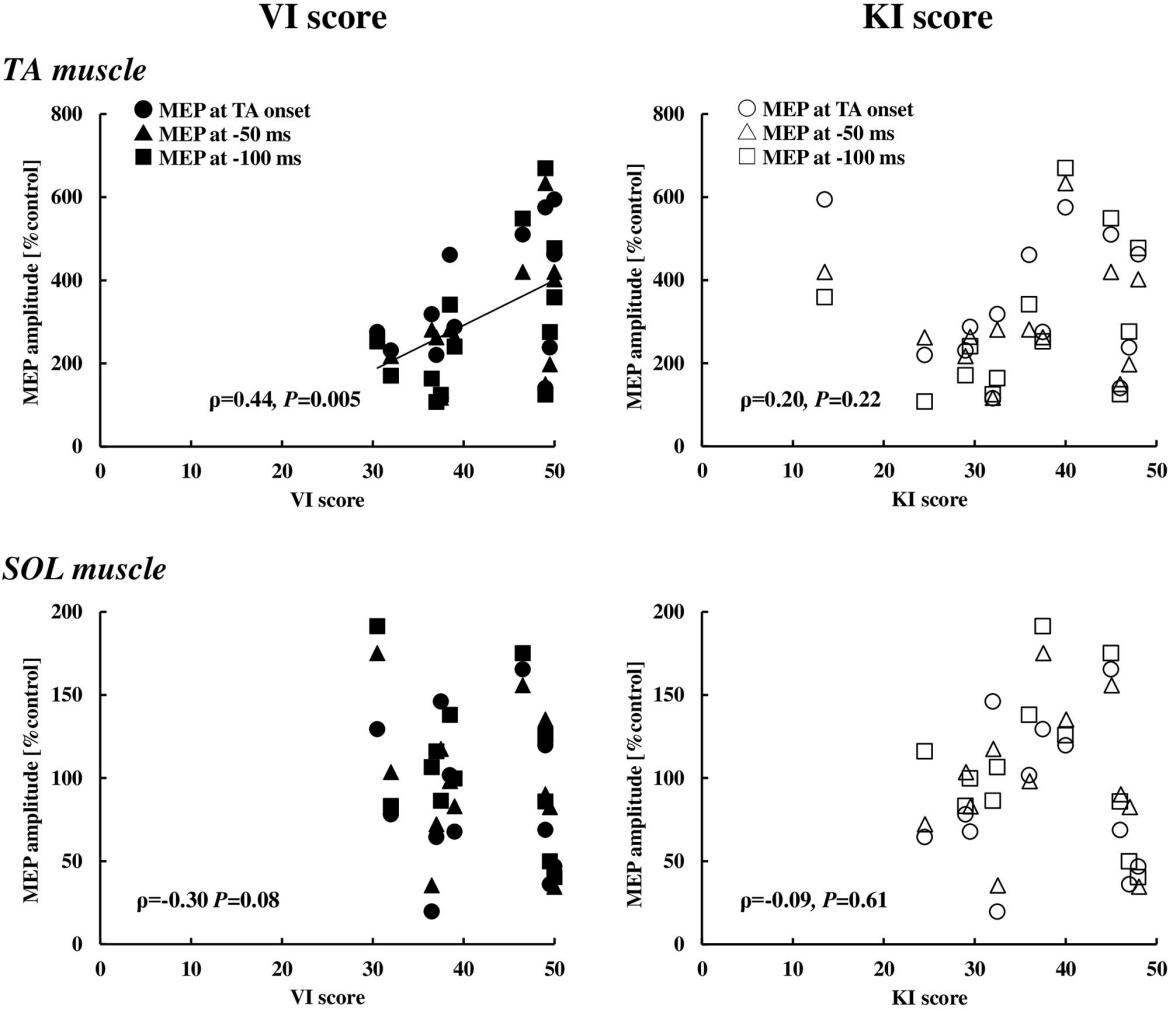
Relationships between the VI or KI score and the MEP in the TA (*n* = 13) and SOL (*n* = 12) muscles at the timings of 0, 50, and 100 ms prior to the EMG onset of the TA muscle. VI, visual imagery; KI, kinesthetic imagery; TA, tibialis anterior; SOL, soleus, MEP, motor evoked potential.

With respect to the motor imagery ability, the VI score (42.9 ± 7.2) was significantly higher compared to the KI score (36.5 ± 10.1) (*p* < 0.01). To explore whether the visual or kinesthetic imagery ability has relation to the MEP enhancement of the TA and SOL muscles, the MEP data at and immediately prior to the TA onset (0 ms, −50 ms, and −100 ms) were pooled and that correlation with the VI or the KI score was analyzed (Figure 6). The VI score, but not the KI score, was significantly correlated to the MEP enhancement in the TA muscle, while a significant correlation was observed neither with the VI score nor the KI score in the SOL muscle.

## Discussion

The major finding from this study was that the corticospinal excitability was significantly increased prior to the EMG activity of the TA muscle during the dart throwing. The ballistic movement of the dart throwing involved a slight elbow flexion followed by a full extension in association with the posterior and anterior movement of the COP. The preceding EMG activity of the TA muscle counteracted the COP changes operating for the incoming elbow flexion and extension, suggesting the contribution of the corticospinal tract of the TA muscle to the APA. Further, the visual, but not kinesthetic, motor imagery ability positively correlated to the MEP enhancement in the TA muscle. To the best of our knowledge, this study provides the first evidence that the corticospinal pathway may play a crucial role for the APA in the lower limb during the ballistic and repetitive throwing movement involving the elbow flexion and extension.

### Kinematic Profiles and Electromyography Activity During the Dart Throwing

Previous studies reported that the APA in the rapid shoulder movements was typically observed from about 100 ms prior to the initiation of the intended movement (Aruin and Latash, 1995b). Rapid shoulder flexion caused the shift of the COG to the anterior direction due to forward shift in the arm placement whereas the COP shifted in a posterior direction in reaction to the movement of the upper limb and the EMG of the postural muscles such as erector spinae, biceps femoris, and the SOL muscles were activated before the initiation of the shoulder flexion. In contrast, the rapid shoulder extension caused the shift of the COG to the posterior direction due to backward shift in the arm placement whereas the COP shifted in an anterior direction in reaction to the movement of the upper limb and the EMG of the postural muscles such as rectus abdominis, rectus femoris, and the TA muscles were activated before the initiation of the shoulder extension. In this study, the dart throwing involved more complex elbow movement, namely, initiated with a slight elbow flexion followed by a full extension (Figure 2) and the COP data also involved both the posterior and anterior shifts. The rapid elbow extension made an anterior shift of the COP drastically, which indicated the posture fell forward eventually. Preceding the elbow movements, therefore, a posterior shift of the COP in advance was most likely to counteract and minimize the incoming anterior shift of the COP accompanied with the rapid elbow extension (Figure 2; Table 1). We could not exclude the possibility that the posterior shift of the COP might play a role in accelerating the whole body in the anterior direction during the elbow extension (Stamenkovic and Stapley, 2016). Nevertheless, by considering the temporal changes in the COP along with the elbow movements, the posterior shift of the COP would accompany the elbow flexion and the following anterior shift of the COP would accompany the elbow extension.

The results of the EMG activity revealed an early onset of the TA muscle and the posterior shift of the COP, i.e., approximately 320 ms prior to that of the agonist TB muscle (Figure 2; Table 1). The APA time calculated from the onset of the EMG or COP was somewhat longer compared to the previous studies by using the traditional simple motor task (Aruin and Latash, 1995b; Petersen et al., 2009), but was similar to a previous study by using the dart throwing task in which the APA time was calculated by the ground reaction force (Juras and Słomka, 2013). The differential APA time among the studies might be attributed to the difference in the task difficulty. In this study, if the TA muscle activity in association with the posterior shift of the COP contributed to the anticipatory postural reactions preceding the ballistic elbow joint movement, one would expect that the timing of the TA onset correlated to that of the COP shift or elbow joint movement. TB muscle is the agonist muscle during the dart throwing, while the BB muscle is considered as the “first muscle activated.” The TA muscle was activated approximately 40 ms prior to the EMG onset of the BB muscle (Figure 4A; Table 1). Interestingly, our results showed that the TA onset correlated to both the onsets of the posterior and anterior shift of the COP and the TA onset correlated to only the onset of the elbow flexion (BB muscle), but not the elbow extension (TB muscle) (Figures 3, 4B). It suggested that the TA muscle activity contributed to the shifts of the COP preceding the elbow extension, while the onset timing varied depending on the elbow flexion. Because the mere rapid elbow flexion involves a shift of the COG in the posterior direction, the COP would have an anterior shift by the APA, if any. Therefore, the posterior shift of the COP associated with the TA muscle activity preceding the dart throwing could not be explained by the APA for the elbow flexion and would be an APA for the incoming anterior shift of the COP along with the elbow extension. In the complex movement of the upper limb such as dart throwing, a series of motions involving a slight elbow flexion followed by an extension might be considered as a preprogrammed “set of motions,” and the TA muscle activity accompanied the posterior shift of the COP might contribute to the APA during the dart throwing, which was probably triggered by the initial slight elbow flexion.

On the other hand, the activation of the SOL muscle might also play a role in the APA for the anterior shift of the COG in association with the ballistic elbow extension (Petersen et al., 2009). In this study, however, the SOL muscle initiated almost simultaneously to the TB muscle, after the onset of the anterior shift of the COP (Figure 2; Table 1), suggesting a compensatory but not anticipatory postural reaction of the SOL muscle. Through this study, it seemed that the SOL muscle was no longer operative for the APA, possibly due to the earlier onset of the TA muscle, which was operating for the posterior shift of the COP and counteracting the incoming anterior shift of the COP associated with the ballistic elbow extension in the dart throwing.

### Corticospinal Excitability During the Anticipatory Postural Adjustment

The results of the MEP in the TA muscle were in line compared to the results mentioned above (Figure 5B). It was no surprise that the MEP was enhanced in both the TA and SOL muscles at the time point of the TB onset because it is known that the EMG activity and increased excitability of the spinal motoneurons contribute to the MEP responses to TMS (Di Lazzaro et al., 1998). On the other hand, the MEPs in the TA muscle significantly increased immediately prior to the onset of the TA muscle, while those in the SOL muscle had no change. The MEP amplitude at −100 ms, −50 ms, and the TA onset, which involved no bEMG activity, exhibited almost the same size compared to the TB onset, which involved bEMG activity, suggesting that it is unlikely that the increased excitability of the spinal motoneuron of the TA muscle contributes mainly to the MEP enhancement prior to the TA onset and it also might be attributed to the increased excitability at a supraspinal level.

Previous studies by using TMS reported that the M1 excitability was modulated before the voluntary movements (Tomberg and Caramia, 1991; Pascual-Leone et al., 1992; Hoshiyama et al., 1996; Chen et al., 1998). The corticospinal excitability of the muscles, which was directly involved in the tasks, increased from about 80–100 ms prior to the EMG onset for the simple reaction time and self-paced movement, respectively (Chen and Hallett, 1999). The postural muscles were not directly involved in the task but activated to minimize the postural displacement from an expected perturbation in advance (Bouisset and Zattara, 1987). In this study, the MEP in the TA muscle at −100 ms, −50 ms, and the TA onset significantly increased compared to the control, suggesting that the MEP in the TA muscle increased from at least 100 ms before the EMG onset of the TA muscle. This is consistent with the results of the previous studies which demonstrated that the corticospinal excitability of the muscles directly involved in the tasks increased from about 80–100 ms before the voluntary movements.

By using TMS combined with H-reflex, Petersen et al. (2009) investigated the modulation of the corticospinal excitability of the SOL muscle during a voluntary heel-raise or handle-pull task and concluded that M1 might be involved in the APA control of the lower limb muscle. Chiou et al. (2016, 2018) by using TMS demonstrated that, when performing the rapid shoulder flexion, the cortical excitability of the erector spinae muscle increased along with the reduced short-interval intracortical inhibition during the APA before receiving any afferent input from the periphery. Massé-Alarie et al. (2018) also examined the corticospinal excitability of the superficial multifidus and rectus abdominis muscles in the preparation of rapid shoulder movements. They concluded that there were two possible mechanisms underlying the motor preparation for the APA: a nonspecific inhibitory mechanism for the superficial multifidus muscle before the Go signal and a task-specific modulation of the corticospinal excitability of the superficial multifidus and rectus abdominis muscles after the Go signal. From the previous studies, the present findings suggested that the corticospinal excitability of the TA muscle increased immediately before the EMG burst (−100 ms–0 ms) at a time window of the APA. It has been suggested that the motoneurons of the TA muscle receive a greater excitatory influence from the M1 compared to the SOL muscle (Brouwer and Ashby, 1992; Bawa et al., 2002), which is known as an antigravity muscle. If it is the case that the stronger the strength of the central control of the muscle, the larger the voluntary drive downstream from the higher brain center (Liang et al., 2011), our result of the longer APA duration of the TA muscle compared with the previous studies might reflect an early modulation by the central command for the postural control.

Taking into account the previous and present results, it suggested that the M1 plays a crucial role in the APA, although several candidates in the cortical and subcortical areas, e.g., SMA, basal ganglia, thalamus, brainstem, vestibule, and spinal cord are also thought to contribute to the APA (Viallet et al., 1992; Jacobs et al., 2009; Ng et al., 2011, 2013). In particular, it has been shown that the APA was impaired in the patients with the lesion of the SMA, while the APA was intact in a patient with a corpus callosum section (Viallet et al., 1992). A 1-Hz repetitive TMS (rTMS), which transiently disrupted SMA, but not in the case of the dorsolateral premotor cortex, resulted in a decreased duration of the APA in both the healthy humans and the patients with Parkinson's disease (Jacobs et al., 2009). A plausible mechanism for the APA is that the enhanced M1 excitability may be attributed to the projections from the SMA, by which the neural circuits responsible for the APA are predecided.

### Cognitive Characteristics and Corticospinal Excitability

Previous studies have reported that the modality dominance of the motor imagery is related to the individual optimal attentional strategy, which was defined by the motor performance outcome under the different focus of the attention conditions (Sakurada et al., 2016, 2017, 2019), namely VI score with the external focus of attention, while the KI score with the internal focus of attention. It is known that the external focus of attention which concentrated on the movement outcome leads to better performance and efficient motor output rather than the internal focus of attention which concentrated on one's body movement (Wulf, 2013). In this study, the VI score was significantly higher compared to the KI score, and there was a significant positive correlation between the MEP in the TA muscle and the VI score, whereas no correlations were found between the MEP in the TA muscle and the KI score or the MEP in the SOL muscle and the VI or KI score (Figure 6). Without any instructions of attentional focus in the present study, although it was unable to identify whether the external or internal focus of attention the participants adopted if any, the results at least suggested that the motor imagery ability for visualizing the imagined movement might refer to the modulation of the corticospinal excitability of the TA muscle and reflect the central motor command during the APA phase.

### Limitations

There are several potential limitations to this study. First, by using the double cone coil, the TMS would spread in the M1 and induce muscle activation not only in the TA and SOL muscles but also in the other muscles (e.g., upper limb and trunk muscles). Therefore, the motor performance of the dart throwing was measured in a separated protocol without TMS (protocol 1), which made it difficult to simultaneously analyze the kinematic and the MEP data in the time course in an identical trial. Second, although 5–10 MEPs were collected at each time point, the number of the MEPs in the final dataset was sometimes less than five at some time points during the motor task (4.9 ± 2.3 on average). Because the trials involving significant bEMG activity in the TA muscle were omitted from the analysis, the relatively small number of trials in these time points and the individuals might increase the variability of the results. Third, the MEP enhancements prior to the TA muscle onset revealed increased excitability of the corticospinal projections to the muscle, but whether the same population of the corticospinal neurons is used for conveying the signal for the APA and that for a voluntary movement involving the TA muscle is unclear. The previous study has referred to the possibility of similar behavior of the MEP between the APA and voluntary movement conditions (Petersen et al., 2009). Finally, because we have not measured the H-reflex or F-wave which reflects the spinal excitability, or the intracortical inhibition or facilitation by means of the paired-pulse TMS which reflects the cortical excitability, we could not assert the underlying mechanisms in the CNS. Taking into account the previous and present results, it is most likely that the increased corticospinal excitability during the APA is attributed to the excitability changes at the supraspinal level such as M1.

### Clinical Applications

Postural instability and impairment of the APA have been shown in the elderly people (Kanekar and Aruin, 2014a,b) and in the patients with CNS disorders, such as stroke (Palmer et al., 1996; Garland et al., 1997; Slijper et al., 2002; Bourke et al., 2015), cerebral palsy (Bigongiari et al., 2011; Girolami et al., 2011), Parkinson's disease (Viallet et al., 1987; Latash et al., 1995), multiple sclerosis (Krishnan et al., 2012; Aruin et al., 2015), and chronic low back pain (Hodges and Richardson, 1996; Massé-Alarie et al., 2012). Therefore, effective rehabilitation interventions, which focus on the APA and for improving postural stability, are needed.

Dart throwing is a coordinated movement of the multi joints and contains the complex elements of the upper limb movements. With such a ballistic movement aiming the dart to the bull's-eye, which involves the slight elbow flexion and almost full extension, is a more intended and goal-directed action. Thus, our findings suggested that a multijoint movement and an intended and goal-directed ballistic movement can induce the longer time of the APA and, therefore, improve the posture control in an efficient way (Aloraini et al., 2019, 2020). In the rehabilitation for the elderly people or the patients with CNS disorders, the great APA might be induced by performing the multi joints movement and more intended and goal-directed action, but not just performing the ballistic upper limb movement. On the other hand, our findings with respect to the individual cognitive characteristics of the KVIQ showed us a possibility that instruction of utilizing the visual motor imagery might lead to further enhancement of the corticospinal excitability for the APA during the ballistic movements.

## Conclusion

This study demonstrates that the corticospinal excitability of the TA muscle increases preceding the ballistic upper limb movement of the dart throwing, suggesting that the corticospinal pathway contributes to the APA in the lower limb in a muscle-specific manner. The extent toward the enhancement of the corticospinal excitability may be related to the visual motor imagery ability of an individual.

## Data Availability Statement

The original contributions presented in the study are included in the article/supplementary material, further inquiries can be directed to the corresponding author/s.

## Ethics Statement

The studies involving human participants were reviewed and approved by Ethics Committee of Kyoto University Graduate School and Faculty of Medicine. The patients/participants provided their written informed consent to participate in this study.

## Author Contributions

AM and NL designed the study. AM, NL, and HU performed the experiment. AM analyzed the data and drafted the first version of the manuscript. AM, NL, and KI interpreted the results. NL and KI edited and revised the manuscript. AM, NL, HU, and KI approved the final version of the manuscript. All authors contributed to the article and approved the submitted version.

## Funding

This research was partially supported by grants from the Japan Society for the Promotion of Science [Grant-in-Aid for Scientific Research (B), 19H03974 to NL] and the Kyoto University internal grant ISHIZUE (to NL).

## Conflict of Interest

The authors declare that the research was conducted in the absence of any commercial or financial relationships that could be construed as a potential conflict of interest.

## Publisher's Note

All claims expressed in this article are solely those of the authors and do not necessarily represent those of their affiliated organizations, or those of the publisher, the editors and the reviewers. Any product that may be evaluated in this article, or claim that may be made by its manufacturer, is not guaranteed or endorsed by the publisher.

## References

[B1] Aloraini S. M. Glazebrook C. M. Pooyania S. Sibley K. M. Singer J. Passmore S. (2020). An external focus of attention compared to an internal focus of attention improves anticipatory postural adjustments among people post-stroke. Gait Posture 82, 100–105. 10.1016/j.gaitpost.2020.08.133 32911092

[B2] Aloraini S. M. Glazebrook C. M. Sibley K. M. Singer J. Passmore S. (2019). Anticipatory postural adjustments during a Fitts' task: comparing young versus older adults and the effects of different foci of attention. Hum. Mov. Sci. 64, 366–377. 10.1016/j.humov.2019.02.019 30856380

[B3] Aruin A. S. Kanekar N. Lee Y. J. (2015). Anticipatory and compensatory postural adjustments in individuals with multiple sclerosis in response to external perturbations. Neurosci. Lett. 591, 182–186. 10.1016/j.neulet.2015.02.050 25711800

[B4] Aruin A. S. Latash M. L. (1995a). The role of motor action in anticipatory postural adjustments studied with self-induced and externally triggered perturbations. Exp. Brain Res. 106, 291–300. 10.1007/BF00241125 8566194

[B5] Aruin A. S. Latash M. L. (1995b). Directional specificity of postural muscles in feed-forward postural reactions during fast voluntary arm movements. Exp. Brain Res. 103, 323–332. 10.1007/BF00231718 7789439

[B6] Barker A. T. Jalinous R. Freeston I. L. (1985). Non-invasive magnetic stimulation of human motor cortex. Lancet 1, 1106–1107. 10.1016/S0140-6736(85)92413-4 2860322

[B7] Bawa P. Chalmers G. R. Stewart H. Eisen A. A. (2002). Response of ankle extensor and flexor motoneurons to transcranial magnetic stimulation. J. Neurophysiol. 88, 124–132. 10.1152/jn.2002.88.1.124 12091538

[B8] Bigongiari A. de Andrade e Souza F. Franciulli P. M. NetoSel R. Araujo R. C. Mochizuki L. (2011). Anticipatory and compensatory postural adjustments in sitting in children with cerebral palsy. Hum. Mov. Sci. 30, 648–657. 10.1016/j.humov.2010.11.006 21453981

[B9] Bouisset S. Zattara M. (1987). Biomechanical study of the programming of anticipatory postural adjustments associated with voluntary movement. J. Biomech. 20, 735–742. 10.1016/0021-9290(87)90052-2 3654672

[B10] Bourke T. C. Coderre A. M. Bagg S. D. Dukelow S. P. Norman K. E. Scott S. H. (2015). Impaired corrective responses to postural perturbations of the arm in individuals with subacute stroke. J. Neuroeng. Rehabil. 12:7. 10.1186/1743-0003-12-7 25605126 PMC4320520

[B11] Brouwer B. Ashby P. (1992). Corticospinal projections to lower limb motoneurons in man. Exp. Brain Res. 89, 649–654. 10.1007/BF00229889 1644127

[B12] Chen R. Hallett M. (1999). The time course of changes in motor cortex excitability associated with voluntary movement. Can. J. Neurol. Sci. 26, 163–169. 10.1017/S0317167100000196 10451737

[B13] Chen R. Yaseen Z. Cohen L. G. Hallett M. (1998). Time course of corticospinal excitability in reaction time and self-paced movements. Ann. Neurol. 44, 317–325. 10.1002/ana.410440306 9749597

[B14] Chiou S. Y. Gottardi S. E. Hodges P. W. Strutton P. H. (2016). Corticospinal excitability of trunk muscles during different postural tasks. PLoS ONE. 11:e0147650. 10.1371/journal.pone.0147650 26807583 PMC4726526

[B15] Chiou S. Y. Hurry M. Reed T. Quek J. X. Strutton P. H. (2018). Cortical contributions to anticipatory postural adjustments in the trunk. J. Physiol. 596, 1295–1306. 10.1113/JP275312 29368403 PMC5878228

[B16] Cohen J. (1988). Statistical Power Analysis for the Behavioral Sciences, 2nd Edn. New York, NY: Routledge Academic.

[B17] Di Lazzaro V. Restuccia D. Oliviero A. Profice P. Ferrara L. Insola A.. (1998). Effects of voluntary contraction on desending volleys evoked by transcranial stimulation in conscious humans. J. Physiol. 508, 625–633. 10.1111/j.1469-7793.1998.625bq.x 9508823 PMC2230886

[B18] Friedli W. G. Hallett M. Simon S. R. (1984). Postural adjustments associated with rapid voluntary arm movements 1. Electromyographic data. J. Neurol. Neurosurg. Psychiatry 47, 611–622. 10.1136/jnnp.47.6.611 6736995 PMC1027860

[B19] Garland S. J. Stevenson T. J. Ivanova T. (1997). Postural responses to unilateral arm perturbation in young, elderly, and hemiplegic subjects. Arch. Phys. Med. Rehabil. 78, 1072–1077. 10.1016/S0003-9993(97)90130-1 9339155

[B20] Girolami G. L. Shiratori T. Aruin A. S. (2011). Anticipatory postural adjustments in children with hemiplegia and diplegia. J. Electromyogr. Kinesiol. 21, 988–997. 10.1016/j.jelekin.2011.08.013 21983006

[B21] Hodges P. W. Richardson C. A. (1996). Inefficient muscular stabilization of the lumbar spine associated with low back pain. A motor control evaluation of transversus abdominis. Spine 21, 2640–2650. 10.1097/00007632-199611150-00014 8961451

[B22] Holm S. (1979). A simple sequentially rejective multiple test procedure. Scand. J. Stat. 6, 65–70.

[B23] Horak F. B. (2006). Postural orientation and equilibrium: what do we need to know about neural control of balance to prevent falls? Age Ageing 35, ii7–ii11. 10.1093/ageing/afl077 16926210

[B24] Hoshiyama M. Kitamura Y. Koyama S. Watanabe S. Shimojo M. Kakigi R. (1996). Reciprocal change of motor evoked potentials preceding voluntary movement in humans. Muscle Nerve. 19, 125–131. 10.1002/(SICI)1097-4598(199602)19:2<125::AID-MUS1>3.0.CO;2-G 8559159

[B25] Jacobs J. V. Lou J. S. Kraakevik J. A. Horak F. B. (2009). The supplementary motor area contributes to the timing of the anticipatory postural adjustment during step initiation in participants with and without Parkinson's disease. Neuroscience 164, 877–885. 10.1016/j.neuroscience.2009.08.002 19665521 PMC2762010

[B26] Juras G. Słomka K. (2013). Anticipatory postural adjustments in dart throwing. J. Hum. Kinet. 37, 39–45. 10.2478/hukin-2013-0023 24146703 PMC3796839

[B27] Kanekar N. Aruin A. S. (2014a). The effect of aging on anticipatory postural control. Exp. Brain Res. 232, 1127–1136. 10.1007/s00221-014-3822-3 24449006 PMC3954907

[B28] Kanekar N. Aruin A. S. (2014b). Aging and balance control in response to external perturbations: role of anticipatory and compensatory postural mechanisms. Age 36, 1067–1077. 10.1007/s11357-014-9621-8 24532389 PMC4082574

[B29] Kasai T. Kawai S. Kawanishi M. Yahagi S. (1997). Evidence for facilitation of motor evoked potentials (MEPs) induced by motor imagery. Brain Res. 744, 147–150. 10.1016/S0006-8993(96)01101-8 9030424

[B30] Kasai T. Taga T. (1992). Effects of varying load conditions on the organization of postural adjustments during voluntary arm flexion. J. Mot. Behav. 24, 359–365. 10.1080/00222895.1992.9941632 14769565

[B31] Kawanishi M. Yahagi S. Kasai T. (1999). Neural mechanisms of soleus H-reflex depression accompanying voluntary arm movement in standing humans. Brain Res. 832, 13–22. 10.1016/S0006-8993(99)01454-7 10375647

[B32] Krishnan V. Kanekar N. Aruin A. S. (2012). Anticipatory postural adjustments in individuals with multiple sclerosis. Neurosci. Lett. 506, 256–260. 10.1016/j.neulet.2011.11.018 22154279

[B33] Latash M. L. Aruin A. S. Neyman I. Nicholas J. J. (1995). Anticipatory postural adjustments during self inflicted and predictable perturbations in Parkinson's disease. J. Neurol. Neurosurg. Psychiatry 58, 326–334. 10.1136/jnnp.58.3.326 7897415 PMC1073370

[B34] Lee W. A. Buchanan T. S. Rogers M. W. (1987). Effects of arm acceleration and behavioral conditions on the organization of postural adjustments during arm flexion. Exp. Brain Res. 66, 257–270. 10.1007/BF00243303 3595773

[B35] Liang N. Nakamoto T. Mochizuki S. Matsukawa K. (2011). Differential contribution of central command to the cardiovascular responses during static exercise of ankle dorsal and plantar flexion in humans. J. Appl. Physiol. 110, 670–680. 10.1152/japplphysiol.00740.2010 21193563

[B36] Malouin F. Richards C. L. Jackson P. L. Lafleur M. F. Durand A. Doyon J. (2007). The Kinesthetic and Visual Imagery Questionnaire (KVIQ) for assessing motor imagery in persons with physical disabilities: a reliability and construct validity study. J. Neurol. Phys. Ther. 31, 20–29. 10.1097/01.NPT.0000260567.24122.64 17419886

[B37] Massé-Alarie H. Flamand V. H. Moffet H. Schneider C. (2012). Corticomotor control of deep abdominal muscles in chronic low back pain and anticipatory postural adjustments. Exp. Brain Res. 218, 99–109. 10.1007/s00221-012-3008-9 22311467

[B38] Massé-Alarie H. Neige C. Bouyer L. J. Mercier C. (2018). Modulation of corticospinal excitability of trunk muscles in preparation of rapid arm movement. Neuroscience. 369, 231–241. 10.1016/j.neuroscience.2017.11.024 29174911

[B39] Massion J. (1992). Movement, posture and equilibrium: interaction and coordination. Prog. Neurobiol. 38, 35–56. 10.1016/0301-0082(92)90034-C 1736324

[B40] Ng T. H. Sowman P. F. Brock J. Johnson B. W. (2011). Premovement brain activity in a bimanual load-lifting task. Exp. Brain Res. 208, 189–201. 10.1007/s00221-010-2470-5 21076820

[B41] Ng T. H. Sowman P. F. Brock J. Johnson B. W. (2013). Neuromagnetic brain activity associated with anticipatory postural adjustments for bimanual load lifting. Neuroimage 66, 343–352. 10.1016/j.neuroimage.2012.10.042 23108270

[B42] Nicholls M. E. Thomas N. A. Loetscher T. Grimshaw G. M. (2013). The Flinders Handedness survey (FLANDERS): a brief measure of skilled hand preference. Cortex 49, 2914–2926. 10.1016/j.cortex.2013.02.002 23498655

[B43] Okubo M. Suzuki H. Nicholls M. E. (2014). A Japanese version of the FLANDERS handedness questionnaire (in Japanese). ShinrigakuKenkyu 85, 474–481. 10.4992/jjpsy.85.13235 25639030

[B44] Palmer E. Downes L. Ashby P. (1996). Associated postural adjustments are impaired by a lesion of the cortex. Neurology. 46, 471–475. 10.1212/WNL.46.2.471 8614516

[B45] Pascual-Leone A. Valls-Solé J. Wassermann E. M. Brasil-Neto J. Cohen L. G. Hallett M. (1992). Effects of focal transcranial magnetic stimulation on simple reaction time to acoustic, visual and somatosensory stimuli. Brain 115, 1045–1059. 10.1093/brain/115.4.1045 1393501

[B46] Petersen T. H. Rosenberg K. Petersen N. C. Nielsen J. B. (2009). Cortical involvement in anticipatory postural reactions in man. Exp. Brain Res. 193, 161–171. 10.1007/s00221-008-1603-6 18956177

[B47] Sakurada T. Hirai M. Watanabe E. (2016). Optimization of a motor learning attention-directing strategy based on an individual's motor imagery ability. Exp. Brain Res. 234, 301–311. 10.1007/s00221-015-4464-9 26466828

[B48] Sakurada T. Hirai M. Watanabe E. (2019). Individual optimal attentional strategy during implicit motor learning boosts frontoparietal neural processing efficiency: a functional near-infrared spectroscopy study. Brain Behav. 9:e01183. 10.1002/brb3.1183 30520270 PMC6346671

[B49] Sakurada T. Nakajima T. Morita M. Hirai M. Watanabe E. (2017). Improved motor performance in patients with acute stroke using the optimal individual attentional strategy. Sci. Rep. 7:40592. 10.1038/srep40592 28094320 PMC5240116

[B50] Slijper H. Latash M. L. Rao N. Aruin A. S. (2002). Task-specific modulation of anticipatory postural adjustments in individuals with hemiparesis. Clin. Neurophysiol. 113, 642–655. 10.1016/S1388-2457(02)00041-X 11976044

[B51] Stamenkovic A. Stapley P. J. (2016). Trunk muscles contribute as functional groups to directionality of reaching during stance. Exp. Brain Res. 234, 1119–1132. 10.1007/s00221-015-4536-x 26746311

[B52] Tomberg C. Caramia M. D. (1991). Prime mover muscle in finger lift or finger flexion reaction times: identification with transcranial magnetic stimulation. Electroencephalogr. Clin. Neurophysiol. 81, 319–322. 10.1016/0168-5597(91)90019-T 1714827

[B53] Viallet F. Massion J. Massarino R. Khalil R. (1987). Performance of a bimanual load-lifting task by parkinsonian patients. J. Neurol. Neurosurg. Psychiatry 50, 1274–1283. 10.1136/jnnp.50.10.1274 3681306 PMC1032450

[B54] Viallet F. Massion J. Massarino R. Khalil R. (1992). Coordination between posture and movement in a bimanual load lifting task: putative role of a medial frontal region including the supplementary motor area. Exp. Brain Res. 88, 674–684. 10.1007/BF00228197 1587326

[B55] Wulf G. (2013). Attentional focus and motor learning: a review of 15 years. Int. Rev. Sport. Exerc. Psychol. 6, 77–104. 10.1080/1750984X.2012.723728

[B56] Yahagi S. Shimura K. Kasai T. (1996). An increase in cortical excitability with no change in spinal excitability during motor imagery. Percept. Mot. Skills 83, 288–290. 10.2466/pms.1996.83.1.288 8873203

